# Consumption, Health Attitudes and Perception Toward Fast Food Among Arab Consumers in Kuwait: Gender Differences

**DOI:** 10.5539/gjhs.v6n6p136

**Published:** 2014-07-15

**Authors:** Abdulrahman O. Musaiger

**Affiliations:** 1 Arab Center for Nutrition, Bahrain

**Keywords:** fast food intake, fast food perception, health attitudes, Kuwait

## Abstract

This study aimed to investigate gender differences in the fast food intake, health attitudes, and perceptions of fast food among adult Arab consumers aged 19 to 65 years in Kuwait. A total of 499 consumers (252 males, 247 females) were selected at convenience from three shopping malls in Kuwait City. The consumers were interviewed using a specially designed questionnaire. The findings revealed that men were more frequently consumed fast food than women (p < 0.001). Men were significantly more likely to consume “double” burgers (52%) than women (29.9%) (P < 0.001). The great majority of consumers (95%) considered fast food harmful to health. However, the consumers were continued to intake fast food (92%), indicating that health information on fast food not necessarly affects their consumption. Local foods were more likely to be considered fast food if eaten as a sandwich or without a disposal container. It can be concluded that fast food perceptions are influenced by gender, media and socio-cultural factors. Nutrition education programmes should focus on nutritive values of the foods rather than on their “fast food” classification.

## 1. Introduction

Fast food has become an important part of the diet of Arab Gulf countries (Bahrain, Kuwait, Oman, Qatar, Saudi Arabia, & United Arab Emirates), and the frequency of fast food intake in these countries is relatively high, especially among adolescents (Al-Hazzaa et al., 2011; Musaiger et al., 2011; Al-Haifi et al., 2013). There are several definitions for fast food, the most common of which refers to food purchased through self-service or carry-out eating places without waiter services (Satia et al., 2004). The rise in the intake of meals prepared outside the home has boosted the consumption of fast food (Dave et al., 2009). Nowadays, fast food is not only eaten the outside home, but also invades the home via the home delivery services provided by almost all fast food restaurants.

Generally, fast foods are characterized as energy-dense, low in micronutrients and fiber, and high in fat, salt, and sugar (Goyal & Singh, 2007). Several studies in Western countries indicated that the frequent intake of fast food was positively associated with obesity (Periera et al., 2005; Niemeir et al., 2006), and negatively associated with fruit and vegetables consumption (Fraser et al., 2010). It was suggested that the time trends in fast food consumption roughly parallel the national time trends in obesity prevalence (Jeffery et al., 2006). Excess body weight and obesity have become the major public health problems in all Arab Gulf countries, with a prevalence ranging from 55% to 82% among adults. It is well documented that the state of Kuwait has one of the highest proportions of overweight and obese adults in the world, with a prevalence of 78% and 82% among men and women respectively (Musaiger, 2011). Lack of intake of fruit and vegetables was also reported in these countries; for example, 81% of Kuwaiti adults consumed less than the recommended daily intake of fruit and vegetables (Musaiger & Al-Hazzaa, 2012).

The high consumption of fast food is a worldwide phenomenon. For instance, 80% of United States people consumed fast food, compared to 67% in New Zealand, 63% in Australia, and 56% in the United Kingdom (Brindal, 2010). In Malaysia, 82% of respondents preferred Western fast food (Nezakati et al., 2011). In Bahrain, 80% of adults consumed fast food (Musaiger et al., 2005). Studies in Arab countries, focused on consumer behavior in relation to fast food, are deficient. Most of these studies focused on the intake of fast food, especially among adolescents (Al-Hazzaa et al., 2011; Musaiger et al., 2011; Al-Haifi et al., 2013), rather than focusing on knowledge, attitudes, and practices with regard to fast food. Several factors were found to be associated with the consumption of fast food, including age, gender, variety, price, delivery services, convenience, location, cleanliness, nutritional value, quality, taste, speed service, and seating capacity (Goyal & Singh, 2007). However, one neglected issue is the consumer’s perception of the definition of fast food, as many consumers view fast food to comprise only Western-style products, despite that, based on the previous definition of “fast food”, many local foods could be considered to be fast food also. The present study, therefore, was carried out to explore the gender differences in fast food eating habits, health attitudes toward fast food, and perceptions regarding the definitions of both Western fast foods and local ready-to-eat foods among adult consumers in Kuwait.

## 2. Methods

Data were based on interviewing the Arab consumers visiting shopping malls in Kuwait City, the capital of Kuwait. Shopping malls have become one of the main places for shopping, eating, and entertainment in the Arab Gulf countries. Three main shopping malls in Kuwait City were selected. Convenience random selection of consumers from these three malls was undertaken; participants were then interviewed by two nutritionists, using a specially designed questionnaire.

An attempt was made to select not fewer than 150 consumers from each shopping mall. The total sample included in this study was 499 (252 males, 247 females). Their ages ranged from 19 to 65 years. Permission to carry out the study was obtained from the administrative section of each mall. A special stand was put in the malls, and the nutritionists asked the consumers whether or not they would like to participate in the survey. Non-responding was not calculated due to the high number of consumers, which made it difficult to count the non-response value. The study was ethically approved by the Nutrition Unit at the Bahrain Center for Studies and Research, as well as by the Arab Center for Nutrition, located in Bahrain.

The questionnaire included three sections: (1) fast food intake, (2) attitudes toward health claims regarding fast food, and (3) perception of the definition of fast food. The first section was based on a validated, previously-used questionnaire (Musaiger et al., 2011). The attitudes section was based on negative health claims regarding fast food, commonly spread via the internet, newspapers, and television in the Arab Gulf countries. The third section included a list of the main Western fast foods and the ready-to-eat foods prepared in self-catering food outlets in Kuwait; the consumers were asked whether they perceived each food as a “fast food” or not.

Data were analysed using the Epi-info statistical software package (CDC, 2013). The Chi-square test was used to determine the association of variables with gender. Satistical significance was set at *p* < 0.05.

## 3. Results

The fast food intakes among adult Arab consumers in Kuwait, classified by gender, are given in Table 1. Nearly 92% of consumers consumed fast food. The weekly frequency intake of fast food was higher among men than women (P < 0.001). Men were significantly more likely to consume “double” burgers (52%) than women (29.9%) (P < 0.001). Although men were more prone to consume large-size French fries (40.9% vs. 34.0%), and soft drinks (37.3% vs. 30.8%) than were women, the differences were not statistically significant. Of consumers, 40% preferred to consume fast food from international chains rather than from local-based businesses.

**Table 1:** Fast food intake among Kuwaiti adult consumers by gender

Practice	Men		Women		*P*-value	Total	
	n	%	n	%		N	%
**Fast food intake day/week**							
Not intake	23	9.1	16	6.5	0.000	39	7.8
1-2	76	30.2	121	49.0		197	39.5
3-4	123	48.8	94	38.0		217	43.5
5+	30	11.9	16	6.5		46	9.2
**Preferred burger size**							
Not intake	23	9.1	16	6.5	0.000	39	7.8
Regular	98	38.9	157	63.6		255	51.1
Double	131	52.0	74	29.9		205	41.1
**Preferred French fries size**							
Not intake	23	9.1	16	6.5	0.093	39	7.8
Regular	126	50.0	147	59.5		273	54.7
Large	103	40.9	84	34.0		187	37.5
**Preferred size of soft drinks intake with fast food meal**							
Not intake	23	9.1	16	6.5	0.201	39	7.8
Small	72	28.6	86	34.8		158	31.7
Regular	63	25.0	69	27.9		132	26.5
Large	94	37.3	76	30.8		170	34.0
**Preferred place for intake of fast food**							
Not intake	23	9.1	16	6.5	0.539	39	7.8
Home (delivery)	34	13.5	44	17.8		78	15.6
Fat food outlet (restaurant)	94	37.3	83	33.6		177	35.5
Both	101	40.1	104	42.1		205	41.1
**Preferred chain of fast food**							
Not intake	23	9.1	16	6.5	0.045	39	7.8
International	91	36.1	110	44.5		201	40.3
Local	37	14.7	20	8.1		57	11.4
Both	101	40.1	101	40.9		202	40.5
**Preparing Western fast food at home**							
Most of time	60	23.8	72	29.1	0.360	132	26.5
Sometimes	137	54.4	121	49.0		258	51.7
Not prepare	55	21.8	54	21.9		109	21.8

Only 5.8% of consumers considered that fast food does not cause harm to health, whereas the remainders perceived either that all fast foods are harmful to health (35.5%), or that some of them (58.7%) are harmful. There was no significant difference between gender regarding this perception (P < 0.061). Men were more likely than women to believe that fast food restaurants are more hygienic compared to other restaurants (44.8% vs. 34.0%; P < 0.028). Nearly two thirds of consumers believed that fast-food intake can cause obesity (73.2%). Women were more likely than men to believe that fast food intake is positively associated with chronic non-communicable diseases (42.9% vs. 34.9%), but the difference was not statistically significant (P < 0.150) (Table 2).

**Table 2:** Attitudes toward health claims on fast foods among Kuwaiti adult consumers by gender

Attitudes	Men		Women		*P*-value	Total	
	N	%	n	%		N	%
**Do you think that fast foods cause harm to general health?**							
Yes, all of them	77	30.6	100	40.4	0.061	177	35.5
Yes, some of them	158	62.7	135	54.7		293	58.7
No	17	6.7	12	4.9		29	5.8
**Do you think that fast foods outlets are more hygienic than other food outlets?**							
Yes	113	44.8	84	34.0	0.028	197	39.5
No	72	28.6	94	38.1		166	33.3
Do not know	67	26.6	69	27.9		136	27.2
**Do you think that meat/chicken used in fast food mixed with unpermitted additives?**							
Yes, in most of fast food	51	20.2	63	25.5	0.374	114	22.8
Yes, in some of fast food	104	41.3	95	38.5		199	39.9
No	97	38.5	89	36.0		186	37.3
**Do you think that regular intake of fast foods cause obesity?**							
Yes	179	71.0	186	75.3	0.557	365	73.2
No	46	18.3	39	15.8		85	17.0
Do not know	27	10.7	22	8.9		49	9.8
**Do you think that regular intake of fast food intake is positively associated with chronic non-communicable diseases?**							
Yes	88	34.9	106	42.9	0.150	194	38.9
No	41	16.3	40	16.2		81	16.2
Do not know	123	48.8	101	40.9		224	44.9
**Do you think that the price of fast food meals is**							
Reasonable	167	66.3	183	74.1	0.056	350	70.1
Expensive	85	33.7	64	25.9		149	29.9

All the Western fast foods were also perceived as fast foods by the consumers, with the highest percentage for burgers (96%) and the lowest for pizza (75%). A significant difference between men and women was reported regarding the perception of sausage sandwiche as fast food (88.1% vs. 80.6%; P < 0.021). As for local foods, it was noted that they were more likely to be perceived as fast food if they were eaten as a sandwich or without a disposal container. For example, sandwiches of *shawarma* (grilled meat/chicken), *falafel* (fried broad beans or chickpeas), and *foul* (boiled broad beans) tended to be perceived as fast foods more often than were foods eaten out of disposal containers, such as rice dishes. Bakery goods and *samosa* (fried vegetable pie) were also more likely to be perceived as fast foods, as generally they are not served on disposal plates. Men were significantly more willing than women to perceive *shawarma* (P < 0.002), *falafel* (P < 0.045), and *foul* (P < 0.036) sandwiches, as well as *samosa* (P < 0.002), as fast foods (Table 3).

**Table 3:** Proportion of perception of Kuwaiti adult consumers to defining commonly consumed food as fast food or non-fast food by gender (which of the following food you considered as fast food?)

Foods	Men (n=252)		Women (n=247)		*P*-value	Total (N= 499)	
	Fast food (%)	Not-fast food (%)	Fast food (%)	Not-fast food (%)		Fast food (%)	Not-fast food (%)
**A. Western foods**							
Burger (all types)	96.0	4.0	98.0	2.0	0.204	97.0	3.0
Pizza	73.4	26.6	76.9	23.1	0.364	75.2	24.8
French fries	92.2	7.8	89.5	10.5	0.183	91.1	8.9
Sausage sandwich	88.1	11.9	80.6	19.4	0.021	84.4	15.6
B. **Local foods**							
Shawarma sandwich (grilled meat/chicken)	81.0	19.0	68.8	31.2	0.002	74.9	25.1
Grilled meat/chicken	16.7	83.3	12.6	87.4	0.193	14.6	88.4
Falafel sandwich (fried broad beans or chickpeas paste)	59.1	40.9	50.2	49.8	0.045	54.7	45.3
Foul sandwich (boiled broad beans)	55.6	44.4	46.2	53.8	0.036	50.9	49.1
Homus (chickpeas paste)	23.4	76.6	18.6	81.4	0.189	21.0	79.0
Matabal (eggplant with sesame paste)	22.6	77.4	17.0	83.0	0.116	19.8	80.2
Tabulah (vegetable salad with pieces of bread)	18.7	81.3	15.0	85.0	0.273	16.8	83.2
Fattayer (bakeries filled with thyme or cheese or meat or condensed yoghurt)	64.5	35.5	58.7	41.3	0.200	61.5	38.5
Samosa(fried vegetable pie)	75.4	24.6	62.8	37.2	0.002	69.1	30.9
Rice dishes (with meat or chicken or fish)	13.1	86.9	8.9	91.1	0.138	11.0	89.0

## 4. Discussion

This study indicated that fast foods were frequently consumed by adult Arab consumers in Kuwait, and that the weekly frequent intake of these foods was significantly higher among men than women. The Arab consumers had a tendency to believe the negative health claims related to fast foods, even though they still consumed these foods. In general, the consumers included in the current study perceived that foods consumed in the form of sandwiches, or those consumed without using a disposal plate, were most likely to be fast foods.

Fast food restaurants have become a global business opportunity, and Kuwait is no exception to this trend. However, nutrition transition, rapid socio-economic changes, and westernization, which have all occurred in Kuwait over the past decades, have significantly influenced the lifestyle and food habits of Kuwaiti consumers. This has resulted in a great change in eating habits, and fast foods have become an essential element in the Kuwaiti diet (Zaghloul et al., 2012). This study confirmed this fact, as the majority of consumers (92%) consumed fast food, and 77% of these consumers also prepared fast food at home. Recently, fast food has become available to consumers in many places, such as shopping malls, airports, universities, hospital cafeterias, petrol stations, parks, and restaurants. Due to the shift from extended to nuclear families, the proportion of people dining out has increased, and in many cases fast food outlets may be the preferred places to dine. This could be due to reasonable price, quick service, convenience, and appropriate environment provided by fast food outlets (Nezakati et al., 2011). It can be noted from this study that the majority of adult consumers (70%) stated that the price of fast food is reasonable, which is mainly due to the high per capita income of Kuwait, as well as the relatively low price of fast food.

This study shows that the proportion of weekly fast food intake by the interviewed participants – i.e., Arab adults in Kuwait – exceeded those reported in both Western (Brindal, 2010) and Asian (Nezakati et al., 2011) countries. Compared with other Arab countries, adult consumers in Kuwait were more likely to consume fast food than their counterparts in Bahrain (92% vs 78%) (Musaiger et al., 2008). The findings of the current study – specifically, that men were more likely to consume fast food than women – is in agreement with similar studies reported in Western countries (Driskell et al., 2006). Consuming larger portion sizes of fast food is another aspect of concern, as fast food restaurants promote larger sizes of these foods at a low cost (Brindal, 2010), which means an increased intake of dietary energy and fat. Cameron-Smith (2002) reported that by paying 12% more to upsize fast food, on average, an Australian consumer may be consuming 23% more energy and 25% more fat. The finding of the current study suggested that men were more willing to consume larger sizes of burgers, French fries, and soft drinks than women. This result is similar to that among young people (15-18 years) in Bahrain (Musaiger et al., 2011).

The link between frequent intake of fast food and occurrence of obesity has been reported (Fraser et al., 2010). The nutritional composition of various fast foods indicated that they are high in calories (Aloia et al., 2013). Therefore, frequent intake of these foods may lead to excess intake of energy, and consequently to excess weight gain. In addition, frequent intake of fast food was found to be associated with low intake of vegetables and fruit (Fraser et al., 2010) and with a sedentary lifestyle (Jeffery et al., 2006). This indicates that consumption of fast food is associated with the risk factors for non-communicable diseases. The findings of the current study revealed that high percentages of consumers perceived that fast food can cause harm to general health, and risk for obesity and non-communicable diseases. Nonetheless, these people continue to consume fast foods. Dave et al. (2009) found that the frequency of fast food intake was not associated with a perceived un-healthfulness of fast food. These researchers concluded that public education regarding the negative health aspects of fast food may not influence its consumption. Satia et al. (2004) have shown that respondents who frequently eat at fast food outlets were perceived various impediments to healthy eating.

The reasons for perceiving a particular food as “fast food” may differ from one community to another. According to Nazakati et al. (2011), conveniences, quick service and self-serving were found to be the main criteria to classify whether a food was “fast food” or not. However, these criteria can be applied to several foods consumed away from home, rather than only Western fast foods. Aloia et al. (2013) reported that Western-style fast foods are still perceived differently from traditional or local foods in India. The result of this study pointed out that the Arab consumers in Kuwait may have different perceptions to Western consumers with regard to whether or not a particular food is “fast food”. Western-style fast foods, and local foods in the form of sandwiches, were more likely to be perceived as fast foods than foods eaten from a disposal plate or container. It seems that among Arab consumers in Kuwait, convenience is the main factor in considering food as “fast food”, irrespective of other factors such as taste, quality, quick service, and cleanness, which were mentioned as factors associated with dining in fast food restaurants (Brindal, 2010). However, more investigation is needed to explore the issue of perceiving whether or not food is “fast food”.

The widespread concept – that Western fast foods have higher contents of energy, fat, and salt than local foods – is not necessarily true. Many local and traditional foods are also rich in fat, salt, and calories. In their nutritional analysis of Western and local fast food consumed in Bahrain, Musaiger and D’Souza (2007) found that many local foods provided by self-catering food outlets had a higher proportion of energy, fat, salt, and cholesterol than Western fast foods. Therefore, nutrition education programmes should focus on the nutritional value of foods rather than whether or not these foods are perceived to be “fast foods”.

Two limitations in this study should be considered. First, the results sample represented Arab people who visited the shopping malls, and therefore it does not necessarily represent the adults in Kuwait in general. However, the only way to obtain a representative sample of adult population in Kuwait is by household sampling. Such methods are difficult to apply, due to political and social reasons. Second, the sample included both Kuwaiti and non-Kuwaiti Arabs; the nationality of the consumers may be a cofounding factor, which in turn could affect the findings. Therefore, it is recommended for future studies to split the consumers into nationals and non-nationals. More information should be included in future studies, especially those related to perception of whether or not foods are “fast foods”. It seems that perceptions regarding the definition of fast food are influenced by gender, socio-cultural factors and the media. However, to the best of our knowledge, this the first study in Arab countries to investigate both the concept and the attitudes toward fast food. We hope that the findings of this study will motivate other researchers to carry out more comprehensive studies.
